# Rapid testing requires clinical evaluation for accurate diagnosis of dengue disease: A passive surveillance study in Southern Malaysia

**DOI:** 10.1371/journal.pntd.0009445

**Published:** 2021-05-20

**Authors:** Chin Fang Ngim, Syed M. Tupur Husain, Sharifah Syed Hassan, Amreeta Dhanoa, Siti Aisyah Abdul Ahmad, Jeevitha Mariapun, Wan Fadhilah Wan Ismail, Nevein Philip Botross Henien, Nowrozy Kamar Jahan, Lian Yih Pong, Hesham Elshahawi, Robert D. Hontz, Tyler Warkentein, Nor’azim Mohd Yunos

**Affiliations:** 1 Jeffrey Cheah School of Medicine and Health Sciences, Monash University Malaysia, Malaysia; 2 United States Naval Medical Research Unit-2, Singapore; 3 Mahmoodiah Health Clinic, Ministry of Health, Malaysia; 4 Faculty of Medicine, University of Malaya, Malaysia; University of Oxford, UNITED KINGDOM

## Abstract

**Background:**

Dengue fever is the most common mosquito-borne infection worldwide where an expanding surveillance and characterization of this infection are needed to better inform the healthcare system. In this surveillance-based study, we explored the prevalence and distinguishing features of dengue fever amongst febrile patients in a large community-based health facility in southern peninsular Malaysia.

**Methods:**

Over six months in 2018, we recruited 368 adults who met the WHO 2009 criteria for probable dengue infection. They underwent the following blood tests: full blood count, dengue virus (DENV) rapid diagnostic test (RDT), ELISA (dengue IgM and IgG), nested RT-PCR for dengue, multiplex qRT-PCR for Zika, Chikungunya and dengue as well as PCR tests for *Leptopspira* spp., Japanese encephalitis and West Nile virus.

**Results:**

Laboratory-confirmed dengue infections (defined by positive tests in NS1, IgM, high-titre IgG or nested RT-PCR) were found in 167 (45.4%) patients. Of these 167 dengue patients, only 104 (62.3%) were positive on rapid diagnostic testing. Dengue infection was significantly associated with the following features: family or neighbours with dengue in the past week (AOR: 3.59, 95% CI:2.14–6.00, p<0.001), cutaneous rash (AOR: 3.58, 95% CI:1.77–7.23, p<0.001), increased temperature (AOR: 1.33, 95% CI:1.04–1.70, p = 0.021), leucopenia (white cell count < 4,000/μL) (AOR: 3.44, 95% CI:1.72–6.89, p<0.001) and thrombocytopenia (platelet count <150,000/μL)(AOR: 4.63, 95% CI:2.33–9.21, p<0.001). Dengue infection was negatively associated with runny nose (AOR: 0.47, 95% CI:0.29–0.78, p = 0.003) and arthralgia (AOR: 0.42, 95% CI:0.24–0.75, p = 0.004). Serotyping by nested RT-PCR revealed mostly mono-infections with DENV-2 (n = 64), DENV-1 (n = 32) and DENV-3 (n = 17); 14 co-infections occurred with DENV-1/DENV-2 (n = 13) and DENV-1/DENV-4 (n = 1). Besides dengue, none of the pathogens above were found in patients’ serum.

**Conclusions:**

Acute undifferentiated febrile infections are a diagnostic challenge for community-based clinicians. Rapid diagnostic tests are increasingly used to diagnose dengue infection but negative tests should be interpreted with caution as they fail to detect a considerable proportion of dengue infection. Certain clinical features and haematological parameters are important in the clinical diagnosis of dengue infection.

## Author summary

With the rise in dengue infection worldwide, dengue fever remains an important diagnosis among patients presenting with fever in the community. We conducted a passive surveillance study in a large community clinic in southern Malaysia. Adults with fever who met the WHO 2009 criteria for probable dengue infection underwent blood tests that were able to detect dengue fever accurately, including rapid diagnostics tests, serology, and molecular tests. Nearly half (45.4%) of the patients were confirmed to suffer from dengue infection in which the widely-used rapid tests were negative in a considerable proportion of dengue-infected patients. This underlines the importance of recognizing clinical and laboratory features suggestive of dengue fever. We found that a history of family or neighbours with dengue infection in the past week, skin rash, a higher temperature, leucopenia (white cell count < 4,000/μL) and thrombocytopenia (platelet count <150,000/μL) were significantly associated with dengue fever whereas runny nose and arthralgia were features that suggest non-dengue conditions. These features in addition to rapid diagnostic kits are useful to guide community based health care workers in the diagnosis of patients presenting with undifferentiated fever.

## Introduction

Dengue fever is the most common mosquito-borne infection in the world. Nearly 2.5 billion people live in dengue endemic regions, and each year an estimated 50–100 million people are reported to have dengue infection, resulting in 500,000 hospitalizations and 20,000 deaths. [1]. These numbers are likely an under-representation as many cases are asymptomatic or remain undiagnosed and unreported due to discrepancies in reporting requirements and inadequate surveillance systems. The actual annual caseload may be closer to 390 million [2].

While all major regions of the world are affected by rising dengue infection rates, nearly 75% of the world’s population who is exposed to dengue lives in the Asia-Pacific region [3]. This region suffers disproportionately from severe dengue, with rates in Southeast Asia 18 times higher than in the Americas [4]. Cases have increased over the last decade in Cambodia, Laos, Malaysia, Singapore, the Philippines and Vietnam [3], with some of these countries experiencing spikes in dengue infection occurring at different periods of time [5]. Changes in the predominant circulating dengue serotypes have also been reported. Malaysia, for example, reported a switch from the predominating serotypes circulating of dengue virus (DENV) DENV-1 and DENV-2 in years 2014–2015 to DENV-2 and DENV-3 in late 2016 [6]. The varying circulating serotypes might affect the detection of dengue fever as studies suggested that the non-structural protein 1 (NS1) rapid diagnostic test could be more sensitive to certain dengue serotypes [7,8].

Public awareness of dengue prevention and control alone is insufficient to reduce the burden of the disease. There needs to be an expanded surveillance system and refinement of epidemiologic and clinical characterization of dengue infections that better informs the healthcare system. Where possible, such surveillance should encompass the dengue-like illnesses such as influenza, chikungunya, Zika, and leptospirosis which are endemic in the population as well. In this study, we conducted a passive surveillance in a community clinic recruiting patients presenting with acute undifferentiated febrile illness where we collect descriptive data in addition to laboratory testing to detect dengue and other viral infections using rapid diagnostic tests (RDT), enzyme-linked immunosorbent assay (ELISA) and polymerase chain reaction (PCR). We described the dengue detection rate using dengue rapid diagnostic tests done at point-of-care testing and explored clinical and laboratory features that distinguish dengue infection from other febrile illnesses in this surveillance study.

## Methods

### Ethics

This study was approved by the Medical Research Ethics Committee, Ministry of Health Malaysia (NMRR-17-2162-37710) and the Monash University Human Research Ethics Committee (Project Number 12078). Written informed consent was obtained from all study participants after explanation of the study objectives. Participation was fully voluntary and all patients’ data were anonymised.

### Study setting

Passive surveillance involving dengue and dengue-like illness was conducted at a community clinic in Johor Bahru with the patient population reflecting the larger community in Malaysia. Johor Bahru is situated in Southern Peninsular Malaysia and is the capital of Johor state which recorded the second highest dengue incidence rate in Malaysia after the state of Selangor, accounting for 13% of all dengue cases in Malaysia for year 2015 and 11% in year 2016 [9]. This six-month cross-sectional study (February to July, 2018) involved outpatients seen at the Fever Clinic of the Mahmoodiah Community Clinic, the largest public health and urban-based clinic in Johor State. The Fever Clinic is a separate wing with three consulting rooms dedicated for patients presenting with fever.

### Study participants and design

Recent trends in Malaysia and countries in this region suggest a shifting age demographic of dengue infections towards adults [10–14], leading to our sampling of adult febrile patients. We calculated the sample size needed to study the false negative rate of dengue RDT based on a study conducted in Brazil where there were 22% false negative samples based on non-structural protein 1 (NS1) testing in which positive dengue cases were identified by using quantitative reverse transcription PCR [15]. According to the 95% confidence interval (CI), the prevalence of false negative samples could go up to 30%. Based on an exact proportion of 0.3, a precision of +/-0.05, confidence interval of 0.95 and an inflation of 10% due to possible rejection rate, the calculated minimum sampling size with sufficient statistical power was 355. Therefore, the calculated minimum sampling size with sufficient statistical power was 355 cases with negative RDT result.

All potential adult patients, aged 18 and above, who presented to the clinic with a history of fever and clinical symptoms suggestive of dengue were screened. Over the six months study period, 368 adult febrile patients who met the World Health Organization (WHO) 2009 criteria for probable dengue virus infection (acute fever and two additional criteria: nausea, vomiting, rash, aches, leukopenia, any warning signs) [16] were included in this study. Patients excluded from the study were those with terminal illnesses or mental illnesses, pregnant mothers, prison inmates and patients incapable of giving consent.

After obtaining informed consent, patients were interviewed by a trained research assistant based on a standardized questionnaire. A comprehensive history which included socio-demographic data, presenting complaint, underlying comorbidities, family history of dengue, neighbourhood and social history were obtained. Following this, height, weight, vital signs and physical examination findings were recorded and 7 milliliters (ml) of blood was drawn from each patient for complete blood count and dengue diagnostic tests using serum separator tubes. Serum was separated from blood and stored at -80°C until further analysis. During the consultation with the clinician, both the RDT and blood count results were available whereas molecular and serological tests were conducted later. The patient was managed accordingly by the clinician. Based on the WHO 2009 guidelines [16], we defined dengue infection in a patient if any of the following diagnostic tests was positive: NSI antigen, IgM (rapid test or ELISA), high-titre IgG on ELISA and viral RNA (nested RT-PCR).

### Laboratory methods

- **Detection of DENV NS1, IgG and IgM using ELISA and lateral flow assays**.

Serological tests were done using the following methods according to the manufacturer’s instructions.

- Dengue Combo Rapid Test-Cassette (Chembio Diagnostics, Inc., NY, USA) to indicate presence of dengue NS1 antigen, dengue IgM or IgG.
- Panbio Dengue IgG Indirect ELISA kit (Abbott) and Panbio Dengue IgM Capture ELISA kit (Abbott) which indicates past dengue and recent dengue infections respectively.
- Panbio Dengue IgG Capture ELISA (Abbott) which detects only high IgG levels (HAI titers ≥ 1:2560) in which a positive test indicates acute secondary dengue infections.
  B **Detection of DENVs by nested RT-PCR**

We performed the nested RT-PCR as previously described by Lanciotti et al. and Klungthong et al. with some modifications [17–19]. Briefly, we extracted RNA from the serum using Maxwell 16 Viral Total Nucleic Acid Purification Kit (Promega). Five μL of viral RNA was converted into cDNA followed by PCR amplification in a single tube by utilizing MyTaq One-Step RT-PCR kit (Bioline) according to kit’s protocol. A pair of primers, D1 and D2 was used in the RT-PCR at the final concentration of 0.125 μM (S1 Table). The RT-PCR reaction was initiated at 45°C for 20 min and then heated at 95°C for 1 min; this was followed by 35 amplification cycles of denaturation at 95°C for 10 s, annealing at 55°C for 10 s and extension at 72°C for 30 s, and a final extension at 72°C for 10 min. The RT-PCR product was subsequently utilized as a PCR template at a dilution of 1:100 in a multiplex PCR amplification which was carried out by using MyTaq Mix (Bioline). The primers, D1, TS1.2013TH, TS2, TS3 and TS4 were used in the multiplex PCR at the final concentration of 0.125 μM. The PCR reaction was initially heated at 95°C for 1 min, followed by 25 amplification cycles of denaturation at 95°C for 15 s, annealing at 55°C for 15 s and extension at 72°C for 30 s, and a final extension at 72°C for 10 min. The PCR product was then analyzed via agarose gel electrophoresis. The positive controls of DENV-1, DENV-2, DENV-3 and DENV-4, which were confirmed by multiplex RT-PCR followed by DNA sequencing [20] and whole genome sequencing (DENV-2, GenBank accession no. MH488959), were identified with the detection of a DNA band at size of 482 bp, 119 bp, 290 bp and 392 bp, respectively [17]. Details of the primers used in nested RT-PCR for DENV detection and serotyping are included in S1 Table.

C **Detection of pathogens by multiplex quantitative real-time reverse transcription PCR (qRT-PCR) and conventional PCR**

We subjected the RNA samples to multiplex qRT-PCR using VIASURE Zika, Dengue & Chikungunya Real-Time PCR Detection Kit (CerTest BIOTEC), which detects Zika, dengue and chikungunya viruses. In order to further identify etiology of dengue-like illnesses, samples negative for dengue, Zika and Chikungunya virus on qRT-PCR (n = 288) were also tested for *Leptopspira* spp., Japanese encephalitis and West Nile virus. The detection of *Leptospira* spp. was performed by multiplex PCR using primers described by Ahmed et al [21] whereas tests for Japanese encephalitis and West Nile virus were performed using conventional RT-PCR with the primer sets described by Parida et al [22] and Lanciotti et. al. [23] respectively.

### Statistical analyses

Categorical variables were summarized by numbers and percentages and continuous variables by the median and interquartile range. Firth’s penalized likelihood logistic regression which reduces small-sample bias in the maximum likelihood estimation was performed to identify demographic factors and clinical and laboratory parameters associated with dengue infection [24,25]. Variables with p values < 0.2 in the univariate analysis with the exception of all warning signs variables (which overlapped with presenting symptoms) were initially considered for the backward selection model. After considering for multicollinearity, the variables haemaglobin and neutrophil count were excluded from the initial model. The presence of skin pallor was not included as well due to no events in the non-dengue group. Categorical variables leucopenia and thrombocytopenia that were derived from the continuous variables, white cell count and platelet count respectively, were retained instead of the latter due to ease of clinical interpretation. We aimed to obtain a parsimonious final model by using a manual backward stepwise approach based on the statistical significance level and the Akaike Information Criteria (AIC) for variable elimination, retaining only variables associated with dengue infection at p < 0.05. All statistical analyses were conducted using Stata 15 (Stata Corp., College Station, TX).

## Results

### Characteristics of study participants

A total of 368 participants were recruited for this study with their socio-demographics and clinical characteristics described in **Table 1**. More than half of the participants were males (n = 227, 62%). The participants’ median age was 30.2 years (range 18 to 78 years). The majority of participants were Malaysians (n = 316, 85.9%), with foreigners making up 14.1% of the study population. Based on the weight and height recorded upon study enrolment, 108 (29.8%) of the participants were obese whereas other co-morbidities reported were less common as seen in **Table 1**. Of the 148 patients with co-morbidity(ies), the majority (n = 114) had a single co-morbidity whereas a smaller number had two (n = 22), three (n = 10) and four co-morbidities (n = 2). Nearly all the participants lived in the Johor Bahru district (n = 366, 99.5%). In addition, 157 (42.7%) had contact with either family members or neighbours who had dengue fever in the previous week prior to enrolment. Sixty-three study participants (17.1%) reported a past history of dengue infection based on information given to them by a medical practitioner in the past.

**Table 1:** Characteristics of the study participants (n = 368).

Characteristics		n (%)
Age (years)	<30	181 (49.2)
	30–45	130 (35.3)
	45–60	48 (13.0)
	>60	9 (2.4)
Gender	Male	227 (61.7)
	Female	141 (38.3)
Nationality	Malaysian	316 (85.9))
	Foreigners*	52 (14.1)
Ethnicity of Malaysians (n = 316)	Malay	216 (68.3)
	Chinese	18 (5.7)
	Indian	71 (22.5)
	Indigenous people	11 (3.5)
Co-morbidity	Obesity	108 (29.3)
	Hypertension	35 (9.5)
	Diabetes mellitus	25 (6.8)
	Asthma	31 (8.4)
Close contact with dengue (past one week)	Yes	157 (42.7)
	No	211 (57.3)
Past history of dengue	Yes	63 (17.1)
	No	305 (82.9)

*Country of origin: Bangladesh (n = 24), China (n = 9), Nepal (n = 8), Pakistan (n = 5), Myanmar (n = 4), Indonesia (n = 1) and India (n = 1).

All the 368 study participants had a history of fever with a median duration of 4 days in which the commonest presenting complaints were fatigue (n = 350, 95.1%), myalgia (n = 318, 86.4%), headache (n = 325, 88.3%) and chills or rigors (n = 304, 82.6%). Interestingly, there was a large proportion of patients (n = 277, 75.3%) who reported runny nose, sore throat or cough which are symptoms usually associated with upper respiratory tract infections. There were 94 (25.5%) subjects with at least one warning sign upon presentation in which the frequency of warning signs were in the following order: restlessness or lethargy (n = 58, 15.8%), abnormal laboratory results depicting increasing haematocrit concurrent with decreasing platelet counts (n = 38, 10.3%), persistent vomiting (n = 31, 8.4%), abdominal pain/tenderness (n = 17, 4.6%), bleeding tendency (n = 14, 3.8%), clinical fluid accumulation (n = 1, 0.3%) and tender hepatomegaly (n = 1, 0.3%). None of the participants had features of severe dengue at study enrolment.

### Results of viral diagnostic tests and rapid diagnostic tests

Of the 368 patients, 167 (45.4%) had dengue infection based on the definition we have described in our methods. In these 167 patients, positivity rates for the respective tests were: NS1 RDT (n = 80, 47.9%), IgM RDT (n = 48, 28.7%), IgM ELISA (n = 62, 37.1%), high titre IgG ELISA (n = 31, 18.6%) and nested RT-PCR (n = 127, 76%). A positive RDT test (either a positive NS1 or IgM or both) was found in 104 (62.3%) dengue patients. Forty subjects who were negative on nested RT-PCR were diagnosed with dengue as they tested positive in the following tests either singly or in combination: IgM RDT (n = 22), IgM ELISA (n = 20), high-titre IgG (n = 11) and NS1 (n = 5). Multiplex qRT-PCR detected dengue in 80 patients however nested RT-PCR were able to detect dengue in a larger cohort of patients (n = 127) which included 79 of the 80 patients detected by qRT-PCR.

Besides DENV, no other pathogens were found in these patients’ serum. Of the 127 samples positive for DENV on nested RT-PCR, 113 (89.0%) were single infections by a dengue serotype. Most of these single infections were DENV-2 (n = 64); followed by DENV-1 (n = 32), and DENV-3 (n = 17); none had DENV-4 mono-infection. Co-infections with two dengue serotypes were noted in the remainder 14 (11.0%) patients: 13 had co-infection with DENV-1 and DENV-2 and one patient had co-infection with DENV-1 and DENV-4.

Of the 167 patients with laboratory confirmed dengue infection, only 104 (62.3%) were positive on point-of-care-testing using the Rapid Diagnostic Test (RDT), determined by either a positive NS1 or IgM or both. Results of the RDT tests which were available to the attending doctor appeared to have influenced clinical diagnosis and decision making as seen in Table 2. When the RDT was positive, 98 (94.2%) were diagnosed with dengue infection in which 33 subjects were admitted to the hospital and 62 patients were managed with daily follow up. On the other hand, for the 63 patients with dengue infection who tested negative on RDT, only eight patients were still provided with a clinical diagnosis of dengue infection by the attending doctor whereas the majority (n = 55) were diagnosed with non-dengue illness. Consequently, for these 63 dengue patients who were RDT negative, 53 patients were managed for other febrile illnesses, 8 were offered follow up and 2 were admitted to the hospital. The dengue infection in these 63 RDT-negative patients was detected based on other laboratory-based tests in the study with some patients testing positive in more than one tests; positivity was found in PCR (n = 47), IgM ELISA (n = 14) and high-titre IgG ELISA (n = 9).

**Table 2:** Comparison of clinical diagnosis and management of all laboratory confirmed dengue infected patients (n = 167) who have positive RDT (n = 104) versus negative RDT test (n = 63).

	Diagnosed with dengue by clinicians		Clinical management plan		
	Yes	No	Admit to hospital	Daily follow up at clinic	Managed as non-dengue illness
RDT positive (n = 104)	98 (94.2%)	6 (5.8%)	33 (31.7%)	62 (59.6%)	9 (8.7%)
RDT negative (n = 63)	8 (12.7%)	55 (87.3%)	2 (3.2%)	8 (12.7%)	53 (84.1%)
Total laboratory confirmed dengue (n = 167)	106 (63.5%)	61 (36.5%)	35 (21.0%)	70 (41.9%)	62 (37.1%)

N.B Percentages were based on rows; denominator is the sum of participants who were either RDT positive or RDT negative

### Dengue infection versus non-dengue infection

As the RDT is not able to detect all dengue infections, clinical and basic laboratory tests such as full blood counts remain essential to differentiate between dengue infections and other febrile illnesses. **Table 3** compares the demographics, clinical presentations and laboratory markers of patients with dengue infection (n = 167) versus those who did not have dengue infection but experienced dengue-like illness (n = 201).

**Table 3:** Clinical demographics and laboratory parameters comparing participants with dengue infection and participants without dengue infection.

		Dengue infection (n = 167)	Non-dengue infection (n = 201)	Unadjusted OR (95%CI)	*p* value
Demographics	Age in years, median (IQR)	31.0 (24.5–40.3)	28.9 (23.6–39.9)	1.00 (0.99–1.02)	0.723
	Male gender	114 (68.3)	113 (56.2)	1.67 (1.09–2.56)	**0.019**
	Foreign nationality	39 (23.4)	13 (6.5)	4.29 (2.22–8.28)	**<0.001**
	Family/neighbour with dengue in past week	96 (57.5)	61 (30.3)	3.08 (2.01–4.73)	**<0.001**
	Past history of dengue	28 (16.8)	35 (17.4)	0.96 (0.56–1.65)	0.877
Co-morbidity	Hypertension	12 (7.2)	23 (11.4)	0.61 (0.30–1.25)	0.179
	Diabetes mellitus	5 (3.0)	20 (10.0)	0.30 (0.11–0.79)	**0.014**
	Asthma	7 (4.2)	24 (11.9)	0.34 (0.15–0.79)	**0.012**
	Obesity	50 (30.1)	58 (29.4)	1.03 (0.66–1.62)	0.885
Presenting symptoms	Duration fever (days), median (IQR)	4.0 (3.0–5.0)	4.0 (3.0–6.0)	0.98 (0.89–1.08)	0.741
	Chills/rigors	141 (84.4)	163 (81.1)	1.26 (0.73–2.17)	0.409
	Fatigue	159 (95.2)	191 (95.0)	1.03 (0.41–2.60)	0.952
	Bone pain	49 (29.3)	70 (34.8)	0.78 (0.50–1.21)	0.266
	Myalgia	141 (84.4)	177 (88.1)	0.74 (0.41–1.33)	0.312
	Arthralgia	116 (69.5)	165 (82.1)	0.50 (0.31–0.81)	**0.005**
	Nausea	100 (59.9)	139 (69.2)	0.67 (0.43–1.02)	0.064
	Vomiting	63 (37.7)	78 (38.8)	0.96 (0.63–1.46)	0.834
	Anorexia	131 (78.4)	143 (71.1)	1.47 (0.91–2.36)	0.114
	Abdominal discomfort/pain	57 (34.1)	92 (45.8)	0.62 (0.40–0.94)	**0.024**
	Diarrhoea	46 (27.5)	61 (30.3)	0.87 (0.56–1.37)	0.560
	Headache	148 (88.6)	177 (88.1)	1.05 (0.56–1.98)	0.877
	Retro-orbital pain	84 (50.3)	104 (51.7)	0.94 (0.63–1.42)	0.783
	Dizziness	117 (70.1)	158 (78.6)	0.64 (0.40–1.02)	0.062
	Runny Nose	58 (34.7)	121 (60.2)	0.35 (0.23–0.54)	**<0.001**
	Sore throat	73 (43.7)	133 (66.2)	0.40 (0.26–0.61)	**<0.001**
	Cough	78 (46.7)	141 (70.1)	0.38 (0.24–0.57)	**<0.001**
	Dyspnoea	32 (19.2)	48 (23.9)	0.76 (0.46–1.25)	0.281
	Bleeding	17 (10.2)	29 (14.4)	0.68 (0.36–1.28)	0.230
	Rash	38 (22.8)	16 (8.0)	3.34 (1.80–6.21)	**<0.001**
Physical examination	Temperature, median (IQR)	36.5 (36.0–37.6)	36.4 (36.0–37.1)	1.21 (0.99–1.48)	0.069
	Pulse rate (per min), median (IQR)	97.0 (85.0–107.0)	98.0 (84.0–110.0)	0.99 (0.98–1.01)	0.284
	Systolic Blood pressure (mmHg), median (IQR)	114.0 (105.0–122.0)	117.0 (108.0–127.0)	0.99 (0.97–1.00)	**0.049**
	Diastolic blood pressure (mmHg), median (IQR)	75.0 (68.0–83.0)	75.0 (68.0–82.0)	1.00 (0.99–1.02)	0.801
	Capillary refill time > 2 sec	4 (1.1)	3 (0.8)	1.56 (0.38–6.41)	0.537
	Dehydrated	10 (6.0)	5 (2.5)	2.38 (0.83–6.83)	0.106
	Pallor	11 (6.6)	0 (0.0)	29.61 (1.73–506.4)	**0.019**
	Rashes on examination	22 (13.2)	8 (4.0)	3.52 (1.55–7.98)	**0.003**
Laboratory tests	Hemoglobin, gm/dL, median (IQR)	15.0 (13.6–16.2)	14.6 (13.4–15.6)	1.13 (1.01–1.26)	**0.028**
	Hematocrit, median (IQR)	44.5 (41.2–47.5)	44.1 (40.5–46.9)	1.03 (0.99–1.07)	0.175
	White cell count x 10^3^, median (IQR)	5.1 (3.3–8.0)	7.7 (5.8–10.1)	0.81 (0.75–0.87)	**<0.001**
	Lymphocyte count, median (IQR)	1.5 (0.9–2.2)	1.9 (1.4–2.5)	0.74 (0.59–0.94)	**0.012**
	Neutrophil count, median (IQR)	2.8 (1.7–4.8)	4.7 (3.4–7.2)	0.76 (0.70–0.84)	**<0.001**
	Platelet count x 10^9^/L, median (IQR)	170 (115–248)	255 (201–311)	0.99 (0.99–0.99)	**<0.001**
	Leukopenia (WCC < 4,000/μL)	61 (36.5)	16 (8.0)	6.49 (3.59–11.75)	**<0.001**
	Thrombocytopenia (Platelet <150,000/μL)	66 (39.5)	17 (8.5)	6.91 (3.87–12.33)	**<0.001**
Warning signs*	WS Abdominal pain/tenderness	13 (7.8)	4 (2.0)	3.83 (1.29–11.38)	**0.015**
	WS Persistent vomiting	18 (10.8)	13 (6.5)	1.73 (0.83–3.60)	0.144
	WS Mucosal bleed	9 (5.4)	5 (2.5)	2.14 (0.73–6.25)	0.163
	WS Lethargy/Restlessness	27 (16.2)	31 (15.4)	1.06 (0.61–1.85)	0.839
	WS Clinical Fluid accumulation	0 (0.0)	1 (0.5)	0.40 (0.02–9.86)	0.574
	WS Tender hepatomegaly	0 (0.0)	1 (0.5)	0.40 (0.02–9.86)	0.574

Values are numbers (percentages) unless stated otherwise; OR = odds ratio; IQR = interquartile range.

* The warning sign “increase in hematocrit concurrent with decreasing platelets” was not included for analysis as blood testing was conducted only once in this study.

In the univariate analysis, clinical and laboratory parameters that were significantly associated with confirmed dengue infections at *p* <0.05, are as follows: gender, foreign nationality, family or neighbour with dengue in past week, diabetes mellitus, asthma, arthralgia, abdominal discomfort or pain, runny nose, sore throat, cough, history of rash, pallor, rashes on examination, haemoglobin count, white cell count, lymphocyte count, neutrophil count, platelet count, leucopenia and thrombocytopenia.

Multiple logistic regression analysis of variables with *p* values < 0.2 in the univariate analysis were conducted and the factors associated with dengue infection are presented in **Table 4**. Dengue infection was significantly associated with with family or neighbours with dengue in the past week (AOR: 3.59, 95% CI:2.14–6.00, p<0.001), cutaneous rash (AOR: 3.58, 95% CI:1.77–7.23, p<0.001), increased temperature (AOR: 1.33, 95% CI:1.04–1.70, p = 0.021), leucopenia (white cell count < 4,000/μL) (AOR: 3.44, 95% CI:1.72–6.89, p<0.001), and thrombocytopenia (platelet count <150,000/μL)(AOR: 4.63, 95% CI:2.33–9.21, p<0.001). Dengue infection was negatively associated with runny nose (AOR: 0.47, 95% CI:0.29–0.78, p = 0.003) and arthralgia (AOR: 0.42, 95% CI:0.24–0.75, p = 0.004). The final model obtained by the multivariable logistic regression analysis has an area under the curve (AUC) of 0.82, 95% CI (0.78–0.87) indicating an acceptable predictive performance of the model. At the optimal cut-off point of -0.28, the model has a sensitivity of 78.4% and specificity of 74.6%. The corresponding Receiver-operating Characteristic (ROC) curve, regression coefficients and equation are presented in S2 Table.

**Table 4:** Multiple logistic regression analysis of factors associated with dengue infection.

		Adjusted Odds Ratio (95%CI)	*p* value
Demographics	Family/neighbour with dengue in past week	3.59 (2.14–6.00)	<0.001
Presenting symptoms	Arthralgia	0.42 (0.24–0.75)	0.004
	Runny Nose	0.47 (0.29–0.78)	0.003
	Rash	3.58 (1.77–7.23)	<0.001
Physical examination	Temperature in centigrade	1.33 (1.04–1.70)	0.021
Laboratory tests	Leucopenia (WCC < 4,000/μL)	3.44 (1.72–6.89)	<0.001
	Thrombocytopenia (Platelet<150,000/μL)	4.63 (2.33–9.21)	<0.001

A sensitivity analysis in which dengue infection is defined based on positive ELISA or nested RT-PCR only is available as a supplementary file (S3 Table). For the univariate analysis (S3A Table), statistical significance for most variables were robust with the exception of abdominal pain/discomfort (p = 0.056), dizziness (p = 0.043) and dyspnea (p = 0.037) and no marked difference was observed with the unadjusted odd ratios for most variables. For the multivariable analysis, similar factors found to be associated with dengue were family or neighbours with dengue in the past week, arthralgia, rash, thrombocytopenia and leucopenia (S3B Table).

## Discussion

Acute undifferentiated febrile illness (AUFI) is a common clinical presentation of symptomatic dengue infection. In a recently published systematic review involving 80,554 cases in South and Southeast Asia, dengue was the most common AUFI, followed by leptospirosis, typhoid, scrub typhus, and influenza [26]. Although WHO 2009 probable case definition of dengue is widely used for clinical decision-making, it lacks specificity because of overlapping clinical features with other AUFI especially during the early phases [27,28]. During our six months prospective surveillance study, 368 adult febrile patients met the WHO 2009 criteria for probable dengue virus infection, of which less than half (45.4%) were confirmed as dengue, based on positivity towards NS1 antigen, nested RT- PCR, IgM or high-titre IgG.

However, if dengue detection was based solely on point-of-care tests using RDT, only 62% (104/167) of these patients would have been detected. In a Cambodian study, the sensitivity of RDT combining NS1 and IgM was only 58% [29]. In a Brazilian study, molecular detection methods detected dengue infection in 33 (22%) out of 150 NS1-antigen negative samples [15]. In our study, of the 264 subjects who were negative on RDT, 63 (23.9%) were diagnosed with dengue using PCR or ELISA tests. This should also serve as a caution to doctors that a negative RDT result does not exclude dengue virus as the causal pathogen. This stresses the need for a simple and convenient criterion based on clinical and hematological findings at the early phases of illness. Based on our study of patients presenting at the community clinic, we identified seven features that could help discern dengue from other non-dengue febrile illness. These were the presence of family or neighbours with dengue in the past one week, cutaneous rash, higher temperature, leucopenia and thrombocytopenia, whereas arthralgia and runny nose were negatively associated with dengue.

Although upper respiratory symptoms (running nose, sore throat, cough) were reported in more than one-third of our patients with laboratory confirmed dengue infection, they were significantly less frequent compared to patients with non-dengue febrile illness. These results concur with other studies suggesting higher frequency of upper respiratory tract infection symptoms among patients with other febrile illness compared to dengue patients [30]. The co-circulation of other respiratory pathogens contributes towards undifferentiated febrile illness in areas where dengue is endemic [27]. Only few patients demonstrated bleeding manifestations and signs suggestive of capillary leakage (prolonged capillary refill, effusions, higher haematocrit levels), with no significant differences between the two groups. This is not an unexpected finding as plasma leakage typically appear after the fourth day of illness [16,31], whereas most of our patients presented early during the course of disease. The presence of rash was significantly higher in dengue patients, concurring with the finding in multiple studies [27,30]. The presence of skin pallor was significantly higher in dengue patients during univariate analysis but was not included in the multivariable analysis due to no events in non-dengue patients. Mittal et al. reported an association of skin paleness with advanced stages of dengue [32], contrariwise most of our patients were seen at early stages of dengue.

Our study findings also indicate that there was no significant difference between the two groups for symptoms commonly associated with dengue fever such as bone pain, myalgia, headache, retro-orbital pain, nausea, vomiting and abdominal discomfort suggesting the poor discriminating ability of these features. In fact, these symptoms were reported more commonly in the non-dengue group. Unlike other studies that found arthralgia positively associated with dengue [27,30], our study demonstrated an inverse relationship. Although an association was found between DENV-3 and arthralgia [33], only 13% of our study patients were infected with DENV-3 which may account for lower frequency of arthralgia amongst our dengue patients. Arthralgia occurs frequently in the course of acute infections with many other viruses, although PCR testing for Zika and chikungunya, which are commonly implicated in arthralgia and arthritis was negative in this study.

Among haematological data, leukocyte and platelets counts, were inversely associated with dengue infections. Leukopenia and thrombocytopenia were seen in 36.5% and 39.5% of dengue patients, compared to <10% of the non-dengue group. Although frequently associated with other viral infections, leukopenia is consistently noted as an independent predictor in dengue diagnosis [27,30] and a decrease in leukocyte count is accompanied by a decrease in platelet count [16]. Although thrombocytopaenia was noted in 39.5% of patients with dengue, only 10.2% had bleeding manifestation. Apart from thrombocytopaenia, bleeding diatheses in dengue patients has multifactorial contributions which include liver impairment, vasculopathy, activation of coagulation and the fibrinolytic system, release of pro-inflammatory cytokines and platelet dysfunction [34].

History of family members or neighbors with dengue was an independent predictor for dengue infections. This finding was supported by previous studies that suggest people gathering with daily activities in a house created the exposure frequency of the bites of dengue-virus infected *Aedes* mosquitoes [35]. Moreover, household with larger numbers of family members were at an added risk for exposure to dengue transmission compared to those with smaller ones [35]. In a study conducted in our neighbouring country, Singapore, foreigners were more likely to be infected with dengue presumably because a large majority of foreign workers live in densely populated quarters with lack of proper facilities whereas in our study, this association was not observed in our adjusted analysis [36].

Several studies have attempted to predict dengue infection based on a constellation of clinical features and laboratory results. A review in 2008 found that patients with dengue had lower platelets, white cell count and neutrophil counts with a higher frequency of petechiae compared to other febrile illnesses. Following that, more studies on predictors of dengue were conducted [37]. For example, in a Puerto Rico study, retro-orbital pain, rash, leucopenia, absence of sore throat and leukopenia were predictive of dengue infection in adults [30]. In another study involving patients presenting early in the course of disease in Brazil, conjunctival redness and leukocyte counts were found to be independent clinical predictors of dengue [25].

The observed variations in clinical features in these studies including ours could be due to differences in age, gender, day of presentation, host immune status, presence of comorbidities and circulating DENV serotypes [34]. Dengue is hyper-endemic in Malaysia with co-circulation of all four DENV serotypes which varies during different time period [11]. During the six months study period, all four serotypes were found to be co-circulating, with predominance of DENV-2 (50.4%) and DENV-1 (25.2%). The predominance of DENV-2 serotypes in our study, could have contributed to lower sensitivity of the RDT, as a study evaluating five diagnostic NS1-based kits showed that sensitivities of these tests significantly decreased with DENV-2 samples [7].

The co-circulation of various DENV serotypes in the similar region facilitates the phenomena of co-infections which accounted for 6% of dengue cases in our study. This was consistent with several studies that have reported co-circulation of different DENV serotypes ranging from 5% to 30% [20]. Although our patients were not followed up to determine outcome, studies have shown that DENV co-infected patients have more severe clinical manifestations [20]. Thus, the knowledge about circulating serotypes is of epidemiological and public health importance.

A number of limitations exist in our study design. This study was conducted at a single community center, which may have limited generalizability of the findings. The center, however, is well visited by the surrounding population and provides a representative sampling of the outpatient population in Malaysia. By recruiting individuals who attended a community clinic, our study population consists of patients presenting at the early stages of disease whose symptoms were likely to be milder. As our study was limited to a single point in time, this precluded the analysis of how clinical and laboratory findings may evolve over time. The primary difficulty in following up with these patients was the outpatient nature of the setting, which made follow-up visits less feasible. Our study was conducted in adults and over a six-month period only which may limit the generalization of our findings as the epidemiology of infectious diseases may differ in other periods of the year. There were 288 subjects who were NS1-negative compared to the original target of 355 patients which may affect the validity of the false negative rate we obtained. Investigations for other pathogens were limited to the 288 samples which tested negative for dengue, chikungunya, and Zika virus on qRT-PCR hence we could not rule out co-infection in the remaining 80 samples. We did not test for typhoid fever or scrub typhus in this study. Bacterial causes of fever which are usually detected through the culture of various body fluids amongst hospitalized patients were not investigated in our community-based study thereby the influence of bacterial co-infection could be underestimated.

The strength of our study was the cross comparative utilization of all possible dengue diagnostic tests: the NS1, IgM, high-titre IgG and PCR, providing a more comprehensive diagnosis of dengue infection. We also tested for a variety of other pathogens using PCR although results were negative. The completeness and accuracy of our data were also assured as this study involved prospective data collection performed by a trained team using a standardized protocol. As dengue studies are largely conducted among hospitalized patients in this region, we hope our study may contribute to a better understanding of dengue at a primary care level.

## Conclusions

As a clinical syndrome, acute undifferentiated febrile illnesses are a diagnostic challenge for clinicians especially during the early phases of illness, as symptoms of dengue overlap with those of other febrile illness. We identified clinical features and useful haematological parameters to enable the differentiation of these two entities. The early distinction between dengue and other febrile conditions may guide the primary care provider towards further diagnostic tests and early treatment, tailored towards the patients. While dengue RDT is increasingly used in the primary care setting to diagnose dengue, we still need to exercise caution in interpreting the RDT negative results. After extensive testing encompassing various diagnostic testing for dengue, in addition to screening serum by PCR for Zika, chikungunya, Japanese encephalitis and West Nile virus as well as *Leptopspira spp*., the cause of AUFI remained undiagnosed in more than 50% of patients. To increase the diagnostic yield, future efforts should include nasopharyngeal sampling to identify potential respiratory pathogens and tests for bacterial co-infections for the etiology of dengue-like illness.

## Supporting information

S1 Checklist STROBE checklist. (DOCX)

S1 Dataset Supporting dataset. (XLS)

S1 Table Primers used in nested RT-PCR for DENV detection and serotyping. (DOCX)

S2 Table Multiple logistic regression model. (DOCX)

S3 Table Sensitivity analysis with reclassification of dengue patients. (DOCX)
