# Supplementary material for: Rapid testing requires clinical evaluation for accurate diagnosis of dengue disease: A passive surveillance study in Southern Malaysia
Source: PLoS Negl Trop Dis. 2021 May 20;15(5):e0009445. doi: 10.1371/journal.pntd.0009445 (PMC8171949; doi:10.1371/journal.pntd.0009445)
Supplement: S1 Table — (DOCX) [file pntd.0009445.s003.docx]

**S1 Table** Primers used in nested RT-PCR for DENV detection and serotyping

| Primer | Sequence (5’ – 3’) | Serotype specificity | Size of PCR product (primers)* |
| --- | --- | --- | --- |
| D1 | tcaatatgctgaaacgcgcgagaaaccg | DENV-1 – 4 | - |
| D2 | ttgcaccaacagtcaatgtcttcaggttc | DENV-1 – 4 | 511 bp (D1 and D2) |
| TS1.2013TH | mgcytcagtgattckagg | DENV-1 | 482 bp (D1 and TS1.2013TH) |
| TS2 | cgccacaagggccatgaacag | DENV-2 | 119 bp (D1 and TS2) |
| TS3 | taacatcatcatgagacagagc | DENV-3 | 290 bp (D1 and TS3) |
| TS4 | ctctgttgtcttaaacaagaga | DENV-4 | 392 bp (D1 and TS4) |

*The size of the PCR product amplified by the serotype-specific primers (TS1.2013TH to TS4) is determined from the priming position of primer D1 in each DENV genome as follows: DENV-1, 118–145; DENV-2, 134–161; DENV-3, 132–159; DENV-4, 136–163
