# Supplementary material for: Rapid testing requires clinical evaluation for accurate diagnosis of dengue disease: A passive surveillance study in Southern Malaysia
Source: PLoS Negl Trop Dis. 2021 May 20;15(5):e0009445. doi: 10.1371/journal.pntd.0009445 (PMC8171949; doi:10.1371/journal.pntd.0009445)

**Supplement 2 Multiple logistic regression model**

**Table A S2 Firth penalized multiple logistic regression model for prediction**

|  |  | Regression coefficient (95%CI) | *p* value |
| --- | --- | --- | --- |
| Demographics | Family/neighbour with dengue in past week | 1.28 (0.76 – 1.79) | <0.001 |
| Presenting symptoms | Arthralgia | -0.86 (-1.44 – -0.28) | 0.004 |
|  | Runny Nose | -0.75 (-1.25 – -0.25) | 0.003 |
|  | Rash | 1.28 (0.57 – 1.98) | <0.001 |
| Physical examination | Temperature in centigrade | 0.29 (0.04 – 0.53) | 0.021 |
| Laboratory tests | Leucopenia (WCC < 4,000/µL) | 1.24 (0.55 – 1.93) | <0.001 |
|  | Thrombocytopenia (Platelet<150,000/µL) | 1.53 (0.85 – 2.22) | <0.001 |
| Intercept |  | -11.00 (-19.94 - -2.06) | 0.016 |

* Regression equation: ln (p/1-p), log odds of dengue = 1.28 x Family/neighbour with dengue (Yes=1) - 0.86 x Arthralgia (Yes=1) - 0.75 x Runny nose (Yes=1) + 1.28 x Rash (Yes=1) + 0.29 x Temperature + 1.24 x Leucopenia (Yes=1) + 1.53 x Thrombocytopenia (Yes=1) -11.00

**Fig A S2 Receiver-operating characteristic (ROC) curve for the multiple logistic model**

Optimal cut-off is -0.28 (Sensitivity = 78.4%; Specificity = 74.6%)


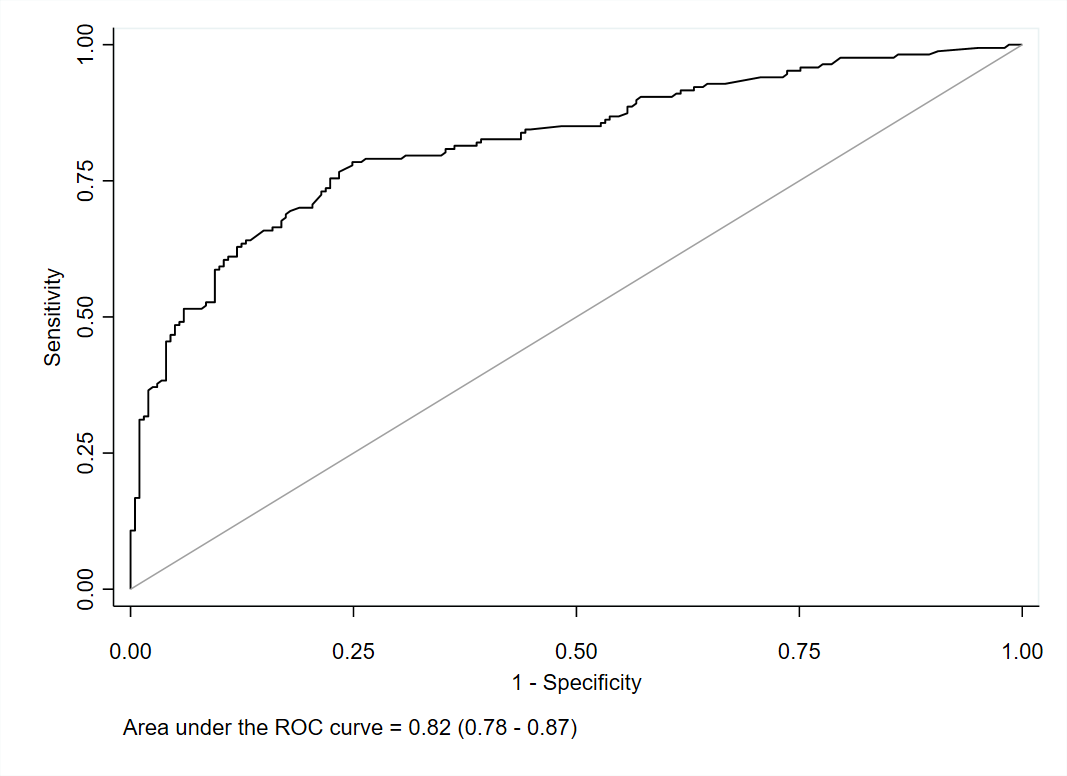

Supplement: S2 Table — (DOCX) [file pntd.0009445.s004.docx]
