# Supplementary material for: Rapid testing requires clinical evaluation for accurate diagnosis of dengue disease: A passive surveillance study in Southern Malaysia
Source: PLoS Negl Trop Dis. 2021 May 20;15(5):e0009445. doi: 10.1371/journal.pntd.0009445 (PMC8171949; doi:10.1371/journal.pntd.0009445)
Supplement: S3 Table — (DOCX) [file pntd.0009445.s005.docx]

**SUPPLEMENT 3 Sensitivity analysis with reclassification of dengue patients**

Sensitivity analysis with reclassification of patients with rapid diagnostic test (RDT) positive but negative for PCR and ELISA as non-dengue patients (n=14) i.e. dengue infection (n=153) and non-dengue infection (n=215)

**Table A in S3** Clinical demographics and laboratory parameters comparing participants with dengue infection (n=153) and participants without dengue infection (n=215)

|  |  | Unadjusted OR (95% CI) * | *p* value |
| --- | --- | --- | --- |
| Demographics | Age in years, median (IQR) | 1.00 (0.98 – 1.01) | 0.592 |
|  | Male gender | 1.58 (1.03 – 2.44) | 0.038 |
|  | Foreign nationality | 4.63 (2.43 – 8.84) | <0.001 |
|  | Family/neighbour with dengue in past week | 3.14 (2.04 – 4.82) | <0.001 |
|  | Past history of dengue | 0.85 (0.49 – 1.47) | 0.551 |
| Co-morbidity | Hypertension | 0.63 (0.30 – 1.31) | 0.218 |
|  | Diabetes mellitus | 0.35 (0.13 – 0.93) | 0.035 |
|  | Asthma | 0.26 (0.10 – 0.68) | 0.006 |
|  | Obesity | 1.04 (0.66 -1.64) | 0.851 |
| Presenting symptoms | Duration fever (days), median (IQR) | 1.00 (0.88 – 1.14) | 0.962 |
|  | Chills/rigors | 1.32 (0.76 – 2.30) | 0.326 |
|  | Fatigue | 0.87 (0.35 – 2.21) | 0.777 |
|  | Bone pain | 0.76 (0.48 -1.18) | 0.220 |
|  | Myalgia | 0.67 (0.37 – 1.22) | 0.193 |
|  | Arthralgia | 0.49 (0.30 -0.79) | 0.004 |
|  | Nausea | 0.67 (0.43 -1.02) | 0.064 |
|  | Vomiting | 0.93 (0.61 – 1.42) | 0.728 |
|  | Anorexia | 1.62 (0.99 – 2.64) | 0.053 |
|  | Abdominal discomfort/pain | 0.66 (0.43 – 1.00) | 0.056 |
|  | Diarrhoea | 0.88 (0.55 – 1.38) | 0.570 |
|  | Headache | 1.09 (0.57 – 2.07) | 0.791 |
|  | Retro-orbital pain | 0.99 (0.66 – 1.50) | 0.972 |
|  | Dizziness | 0.62 (0.38 – 0.99) | 0.043 |
|  | Runny Nose | 0.36 (0.23 – 0.55) | <0.001 |
|  | Sore throat | 0.45 (0.29 – 0.68) | <0.001 |
|  | Cough | 0.36 (0.23 – 0.55) | <0.001 |
|  | Dyspnoea | 0.57 (0.34 – 0.97) | 0.037 |
|  | Bleeding | 0.66 (0.34 – 1.25) | 0.201 |
|  | Rash | 3.02 (1.66 – 5.49) | <0.001 |
| Physical examination | Temperature, median (IQR) | 1.16 (0.95 – 1.42) | 0.145 |
|  | Pulse rate (per min), median (IQR) | 0.99 (0.98 – 1.00) | 0.205 |
|  | Systolic Blood pressure (mmHg), median (IQR) | 0.99 (0.97 – 1.00) | 0.065 |
|  | Diastolic blood pressure (mmHg), median (IQR) | 1.00 (0.99 – 1.02) | 0.618 |
|  | Capillary refill time > 2 sec | 1.83 (0.45-7.50) | 0.403 |
|  | Dehydrated | 2.80 (0.98 – 8.03) | 0.055 |
|  | Pallor | 10.46 (1.87 – 58.66) | 0.008 |
|  | Rashes on examination | 4.18 (1.84 – 9.47) | 0.001 |
| Laboratory tests | Hemoglobin, gm/dL, median (IQR) | 1.14 (1.02 – 1.27) | 0.020 |
|  | Hematocrit, median (IQR) | 1.03 (0.99 – 1.07) | 0.141 |
|  | White cell count x 10^3^, median (IQR) | 0.78 (0.73 – 0.84) | <0.001 |
|  | Lymphocyte count, median (IQR) | 0.65 (0.51 – 0.84) | 0.001 |
|  | Neutrophil count, median (IQR) | 0.74 (0.67 – 0.82) | <0.001 |
|  | Platelet count x 10^9^/L, median (IQR) | 0.99 (0.99 – 0.99) | <0.001 |
|  | Leukopenia (WCC < 4,000/µL) | 7.34 (4.08 – 13.19) | <0.001 |
|  | Thrombocytopenia (Platelet <150,000/µL) | 6.69 (3.83 – 11.68) | <0.001 |
| Warning signs | WS Abdominal pain/tenderness | 3.38 (1.21 – 9.43) | 0.020 |
|  | WS Persistent vomiting | 1.35 (0.65 – 2.80) | 0.414 |
|  | WS Mucosal bleed | 1.88 (0.66 – 5.34) | 0.234 |
|  | WS Lethargy/Restlessness | 0.92 (0.52 – 1.62) | 0.761 |
|  | WS Clinical Fluid accumulation | 0.47 (0.02 – 11.51) | 0.641 |
|  | WS Tender hepatomegaly | 0.47 (0.02 – 11.51) | 0.641 |
|  | WS Laboratory result increasing hematocrit & decreasing platelets | 10.64 (4.20 – 26.94) | <0.001 |

*Firth penalized odds ratio; values are numbers (percentages) unless stated otherwise; OR = odds ratio; IQR = interquartile range.

**Table B in S3** Firth Penalized multiple logistic regression analysis of factors associated with dengue infection

|  |  | Adjusted Odds Ratio (95% CI) * | *p* value |
| --- | --- | --- | --- |
| Demographics | Family/neighbour with dengue in past week | 3.16 (1.88 – 5.31) | <0.001 |
|  | Foreign nationality | 2.58 (1.17 – 5.67) | 0.018 |
| Presenting symptoms | Arthralgia | 0.46 (0.26 – 0.82) | 0.008 |
|  | Cough | 0.52 (0.31 – 0.87) | 0.014 |
|  | Rash | 3.54 (1.77 – 7.06) | <0.001 |
| Laboratory tests | Leucopenia (WCC < 4,000/µL) | 4.36 (2.18 – 8.71) | <0.001 |
|  | Thrombocytopenia (Platelet<150,000/µL) | 3.68 (1.88 – 7.18) | <0.001 |

*Firth penalized odds ratio
